# Sensing Characteristics of Flame-Spray-Made Pt/ZnO Thick Films as H_2_ Gas Sensor

**DOI:** 10.3390/s90906652

**Published:** 2009-08-26

**Authors:** Nittaya Tamaekong, Chaikarn Liewhiran, Anurat Wisitsoraat, Sukon Phanichphant

**Affiliations:** 1 Nanoscience Research Laboratory, Department of Chemistry, Faculty of Science, Chiang Mai University, Chiang Mai, 50200, Thailand; E-Mail: doramon_koygy@hotmail.com (N.T.); 2 Department of Physics, Faculty of Science, Chiang Mai University, Chiang Mai, 50200, Thailand; E-Mail: chaikarn_l@yahoo.com (C.L.); 3 National Electronics and Computer Technology Center, Pathumthani, 12120, Thailand; E-Mail: anurat.wisitsoraat@nectec.or.th (A.W.)

**Keywords:** Pt/ZnO nanoparticles, flame spray pyrolysis, H_2_, gas sensor

## Abstract

Hydrogen sensing of thick films of nanoparticles of pristine, 0.2, 1.0 and 2.0 atomic percentage of Pt concentration doped ZnO were investigated. ZnO nanoparticles doped with 0.2–2.0 at.% Pt were successfully produced in a single step by flame spray pyrolysis (FSP) technique using zinc naphthenate and platinum(II) acetylacetonate as precursors dissolved in xylene. The particle properties were analyzed by XRD, BET, SEM and TEM. Under the 5/5 (precursor/oxygen) flame condition, ZnO nanoparticles and nanorods were observed. The crystallite sizes of ZnO spheroidal and hexagonal particles were found to be ranging from 5 to 20 nm while ZnO nanorods were seen to be 5–20 nm wide and 20–40 nm long. ZnO nanoparticles paste composed of ethyl cellulose and terpineol as binder and solvent respectively was coated on Al_2_O_3_ substrate interdigitated with gold electrodes to form thin films by spin coating technique. The thin film morphology was analyzed by SEM technique. The gas sensing properties toward hydrogen (H_2_) was found that the 0.2 at.% Pt/ZnO sensing film showed an optimum H_2_ sensitivity of ∼164 at hydrogen concentration in air of 1 volume% at 300 °C and a low hydrogen detection limit of 50 ppm at 300 °C operating temperature.

## 1. Introduction

Semiconductor sensors are widely used for gas sensing. One research interest in this field is the search for materials that exhibit high sensitivity and fast response times. Recently, semiconducting nanostructures have earned attention due to their huge surface-to-volume ratios. In general, a gas sensor’s performance is highly dependent on the sensor material’s surface area. Gas sensors based on semiconducting metal oxide nanostructures are expected to exhibit better sensing properties than gas sensors based on bulk or thin films [1]. However, the band-gap energy of semiconductor is directly related to its preparation method. Wang and coworkers [2] reported that size and composition induced band-gap change in nanostructured compound of II–VI semiconductors i.e. when the particle size decreased, the band-gap energy increased.

ZnO is an interesting chemically and thermally stable *n*-type semiconductor of wurtzite structure with a large-band gap energy of 3.37 eV at low temperature and 3.3 eV at room temperature [3–6], and with high sensitivity to toxic and combustible gases. It is sensitive to many gases at moderate temperature, especially H_2_ gas [7–12]. For comparison with the same doping material there were many reports of other preparation methods. Xu *et al*. [7] prepared pure ZnO powder by chemical precipitation. The response characteristics of 0.5 wt% Pt/ZnO nanoparticles to 0.2% H_2_ in air at 330 °C was reported with the sensitivity of 2. Moreover, Rout *et al*. [9] reported ZnO nanowires/nanotubes prepared by electrochemical deposition on alumina membranes. The response characteristics for 1 at.% Pt/ZnO nanoparticles were obtained. The sensitivity of ZnO nanowires without and with Pt impregnation for 1,000 ppm of H_2_ were 43 and 825 at 150 °C, respectively, indicating the enhancement in sensitivity by incorporating Pt into the ZnO nanowires. The response time for the as-prepared and the Pt-impregnated ZnO were 54 and 42 s respectively with the recovering times of 5 and 4 s respectively. In addition, Wang *et al*. [10] reported ZnO nanorods deposited by Molecular Beam Epitaxy (MBE) and detection of hydrogen at room temperature with catalyst-coated multiple ZnO nanorods. The Pt metal catalyst coating deposited on multiple ZnO nanorods were compared for their effectiveness in enhancing sensitivity for detecting hydrogen at room temperature. Pt-coated nanorods showed a relative response of up to 8% in room temperature resistance upon exposure to a hydrogen concentration in N_2_ of 500 ppm. Tien *et al*. [11] reported that ZnO nanorods were deposited by Molecular Beam Epitaxy (MBE) and hydrogen sensing at room temperature with Pt-coated ZnO thin films and nanorods. A comparison of the sensitivities was reported for detecting hydrogen with Pt-coated single ZnO nanorods and thin films. The Pt-coated single nanorods showed a current response of approximately a factor of 3 larger at room temperature upon exposure to 500 ppm H_2_ in N_2_ than the thin films of as-prepared ZnO. For comparison with other doping materials, Xu *et al*. [7] reported that a Ru-doped ZnO gas sensor showed the selectivity of 0.2% H_2_ at 230 °C and 400 °C were 6 and 2, respectively. The Rh-doped ZnO gas sensor had good gas selectivity for 0.2% H_2_ of 115 at 300 °C. A Ag-doped ZnO gas sensor showed characteristic response of 9 for 0.2% H_2_ at 400 °C. A summary comparing H_2_ sensing with metal-doped ZnO prepared by several synthetic methods is shown in Table 1. ZnO is one of the most widely applied oxide-gas sensor. ZnO gas sensing materials owe to their high chemical stability, low cost, and good flexibility in fabrication. Various types of ZnO-based gas sensors, such as single crystal [13–15], sintered pellet [16,17–19], heterojunctions, thin film [18,20–22], and thick films [4,5,23–25], were demonstrated. The application of nanomaterials to the design of hydrogen gas sensor was nowadays one of the most active research fields, due to their high activity, high surface-to-bulk ratio, good adsorption characteristics and high selectivity. The gas sensing mechanism involves chemisorptions of oxygen on the oxide surface followed by charge transfer during the reaction between chemisorbed oxygen reducing and target gas molecules. However, the physical and sensing properties of semiconductor gas sensors are directly related to their preparation e.g. particle size, sensing film morphology, crack surface [22], and film thickness [4,26–34] as well as sensing film characteristics.

Flame spray pyrolysis (FSP) is a promising technique for the synthesis of high purity nano-sized materials with controlled size and crystallinity in a single step. It was systematically investigated using an external-mixing gas-assisted atomizer supported by six premixed methane–oxygen flamelets [35]. With decreasing oxidant flow rate, specific surface area increased as the spray flame length was reduced leading to shorter residence time allowing less time for particle growth. Using oxygen as oxidant,the droplets burn much faster than in air. Thus product particles experience longer residence times at higher temperature causing lower specific surface area. Therefore the specific surface area of the nanoparticles can be controlled by adjusting the oxidant flow rates. After evaporation and combustion of precursor droplets, nanoparticles were formed by nucleation, condensation, coagulation, coalescence and Pt formation deposited on the ZnO support. The effect of Pt dopant on the specific surface area of the nanoparticles and crystalline sizes were also investigated [35,36].

The addition of noble metal to semiconducting oxide is known to be an effective mean to enhance detection of specific gases. Platinum (Pt) is found to be the most effective catalyst for H_2_ gas [7–12]. There have been no previous studies on the gas-sensing behaviors of Pt/ZnO nanoparticles synthesized by FSP. Therefore, FSP synthesis of ZnO and Pt/ZnO nanoparticles and their H_2_ gas-sensing properties are studied in this work.

## 2. Experimental

### 2.1. Particles Synthesis and Characterization

Zinc naphtenate (Aldrich, 8 wt% Zn) and platinum (II) acetylacetonate [Pt (acac)_2_, Aldrich, 97% Pt] were used as precursors because the zinc naphtenate had a high heat of combustion and Pt(acac)_2_ dissolves well in xylene, which also has a high heat of combustion. The precursors were dissolved in xylene (Carlo Erba, 98.5%) to obtain a 0.5 mol/L precursor solution. In a typical run, the precursor was fed into a FSP reactor by a syringe pump with a rate of 5 mL/min while 5 L/min O_2_ is being dispersed (5/5 flame). The gas flow rates of methane and O_2_ supporting flamelets were 1.19, and 2.46 L/min, respectively. The pressure drop at the capillary tip was kept constant at 1.5 bars by adjusting the orifice gap area at the nozzle.

**Figure 1.**
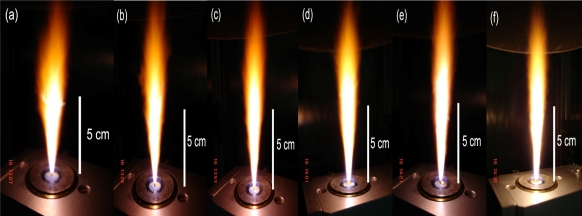
Spray flame (0.5 M zinc naphtenate and Pt (acac)_2_ in xylene) of (a) pure ZnO, (b–f) 0.2–2.0 at.% Pt/ZnO nanoparticles producing 5 mL/min of liquid precursor feed rate and dispersed by O_2_ (5 L/min) at 1.5 bar pressure drop across the nozzle tip. The flame heights were observed ranging from 10–12 cm with slight increasing the combustion enthalpy and Pt concentration.

The flame height was observed to be approximately 10–12 cm, and was increased slightly by increasing the combustion enthalpy. The combustion enthalpies are directly dependent on the particular solvent, starting materials and dopants. All samples showed a yellowish-orange flame as seen in Figure 1. The temperatures for the spray flame were typically in the range of 2,000 K to 2,500 K [37]. The liquid precursor mixture was rapidly dispersed by a gas stream and ignited by a premixed methane/oxygen flame. After evaporation and combustion of precursor droplets, particles are formed by nucleation, condensation, coagulation, coalescence, and Pt deposited on ZnO support. Finally, the nanoparticles were collected on glass microfibre filters with the aid of a vacuum pump. Undoped ZnO nanopowder was designated as P0 while the ZnO nanopowders doped with 0.2–2.0 at.% Pt were designated as P1–P5, respectively. Powders of the various ZnO samples were characterized by X-ray diffraction (XRD) and the specific surface area (*SSA*_BET_) of the nanoparticles was measured by nitrogen adsorption (BET analysis), scanning electron microscopy (SEM) and transmission electron microscope (TEM).

### 2.2. Sensing Films Preparation and Characterization of the Gas Sensing Properties

Sensing films were prepared by mixing the nanoparticles into an organic paste composed of ethyl cellulose and terpineol, which acted as a vehicle binder and solvent, respectively. The resulting paste was spin-coated on Al_2_O_3_ substrates with predeposited interdigitated Au electrodes. The films were then annealed at 400 °C for 2 h (with heating rate of 2 °C/min) for binder removal. The particle size of films was grown slightly after the films were annealed at 400 °C. The fabricated sensors using P0, P1, P3, and P5 powders were now labeled as S1, S2, S3, and S4, (for Figures 6 and 7 labeled as S0, S1, S2 and S3) respectively. The morphology and the cross section of sensing films were analyzed by SEM.

The gas-sensing characteristics of metal oxide nanoparticles were characterized towards H_2_. The flow through technique was used to test the gas-sensing properties of thin films. A constant flux of synthetic air of 2 l/min was mixed with desired concentrations of pollutants. All measurements were conducted in a temperature-stabilized sealed chamber at 20 °C under controlled humidity. The external NiCr heater was heated by a regulated dc power supply to different operating temperatures. The operating temperature was varied from 200 °C to 350 °C. The resistances of various sensors were continuously monitored with a computer-controlled system by voltage-amperometric technique with 5 V dc bias and current measurement through a picoammeter. The sensor was exposed to a gas sample for ∼5 minutes for each gas concentration testing and then the air flux was restored for 15 minutes. The H_2_ concentration was varied from 200 to 1% in volume percentage of concentration.

## 3. Results and Discussion

### 3.1. Particles Properties

**Figure 2.**
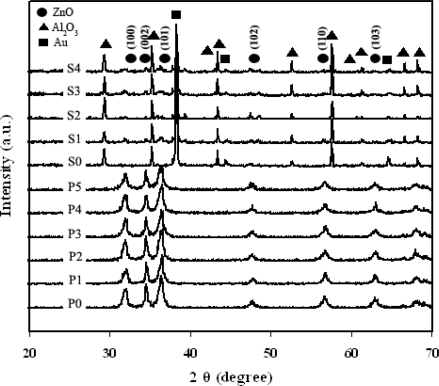
XRD patterns of flame-spray-made (5/5) 0.2–2 at.% Pt/ZnO as-prepared (P0–P5), Au/Al_2_O_3_ substrate (S0), and samples P0, P1, P3, and P5 were spin-coated on Au/Al_2_O_3_ substrate after annealing and sensing test (S1, S2, S3, and S4) [(•) ZnO; (▴) Al_2_O_3_; (▪) Au].

Figure 2 shows the XRD patterns of samples as-prepared (P0–P5), Au/Al_2_O_3_ substrate (S0), and sensors after annealing (S1, S2, S3 and S4).The samples P0–P5 were highly crystalline, and all peaks can be confirmed to be the hexagonal structure of ZnO (Inorganic Crystal Structure Database [ICSD] Coll. Code: 067454 [38,39]). The diffraction patterns of Al_2_O_3_ (ICSD Coll. Code: No. 085137 [38,40]) (filled diamonds) and Au (ICSD Coll. CAS No. 7440-57-5 [38,41]) (filled rectangular) from the substrates are also visible in the S0, S1, S2, S3, and S4. Variation in intensity is due to change in texturization of the crystal plane orientation while preparing sensing films. (data for S1-S4 are XRD from sensing film coated on Au/Al_2_O_3_ substrates while data for P0-P5 are purely powder XRD).

**Figure 3.**
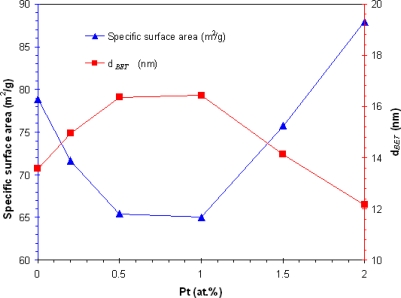
The specific surface area (*SSA*_BET_), *d*_BET_ of ZnO and 0.2–2.0 at.% Pt/ZnO nanoparticles

The average BET equivalent particle diameter (*d*_BET_) were calculated using the average of the density of ZnO and Pt/ZnO taken into account for their weight content of different doping as shown in Figure 3, *SSA*_BET_ increased while the *d*_BET_ decreased with increasing Pt concentration from 1 to 2 at.%. When Pt particles were formed and deposited on the ZnO support processes in the flame, the Pt created a new nucleation center, which in turn changed the nucleation type from homogeneous to heterogeneous, and deteriorated the deposition forming leading to the agglomeration of the tiny Pt particles at high doping level. Pt doping did not affect the ZnO grain size, crystallinity, and the particle morphology. The FSP afforded small Pt particles attached to the surface of the supporting ZnO nanoparticles, indicating a high *SSA*_BET_. The larger crystallite diameters indicate clumping and clusters of Pt, translating into a poor dispersion of the Pt nanoparticles on ZnO support which affected to the decreasing of the *SSA*_BET_. Thus, the agglomerated Pt particles were formed seriously with higher doping level leading to the larger Pt particles. The larger particles have a low *SSA*_BET_. On the other hand, the smaller particles have a high *SSA*_BET_, thus it had many area to react with the molecule of the analyte gas. It can be assumed that the decreasing of the *SSA*_BET_ affected to decrease the depletion layer at the surface, thus the reaction of reducing gas H_2_ with the charged oxygen species destroys the electron localization process drastically reduced and a change in conductivity was decreased. Thus, the poor-dispersed of Pt should be shown the lower sensing performance than the well-dispersed of Pt. Accurate particles size and morphology of ZnO and Pt dispersion were confirmed by SEM and TEM images.

**Figure 5.**
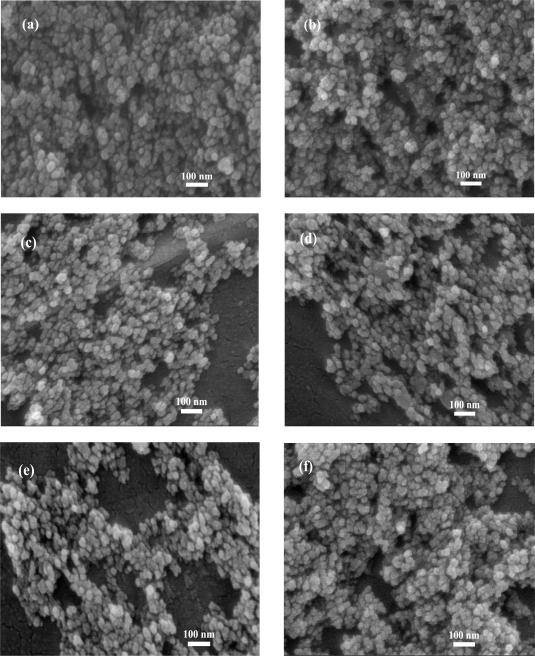
SEM micrographs of highly crystalline flame-made (5/5) (a) pure ZnO (P0) and doped with (b) 0.2 at.% Pt (P1), (c) 0.5 at.% Pt (P2), (d) 1.0 at.% Pt (P3), (e) 1.5 at.% Pt (P4) and (f) 2.0 at.% Pt (P5) nanoparticles for SEM analysis.

Figure 5 shows the morphology of highly crystalline flame-made (5/5) (a) pure ZnO (P0), (b) 0.2 at.% Pt/ZnO (P1), (c) 0.5 at.% Pt/ZnO (P2), (d) 1.0 at.% Pt/ZnO (P3), (e) 1.5 at.% Pt/ZnO (P4) and (f) 2.0 at.% Pt/ZnO (P5) nanoparticles for SEM analysis. SEM micrographs in Figure 5(a) show the nanospheres with an average diameter of 10–20 nm, and several nanospheres connected with each other. From this observation, it was found that the rough morphology and the rough particle sizes were not changed with increasing Pt doping levels. Nevertheless, the accurate sizes and morphology of the nanoparticles can be estimated from the TEM analysis. While the SEM images provide 3-D morphology and estimated particle sizes, TEM images can reveal internal structure and a more accurate measurement of particle size and morphology.

**Figure 6.**
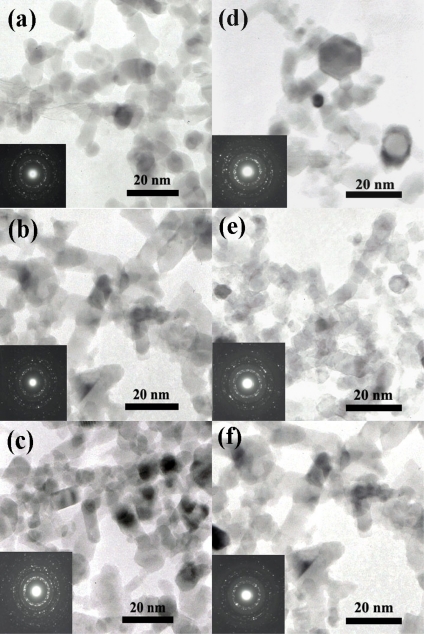
TEM images of ZnO (a), 0.2 (b), 1.0 (c) and 2.0 (d) at.% Pt doped ZnO nanoparticles. ZnO particle sizes and morphology were spheroidal, hexagonal and rod-like.

Figure 6(a)–(f) shows transmission electron microscope (TEM) images of ZnO, 0.2, 0.5, 1.0, 1.5 and 2.0 at.% Pt doped ZnO nanoparticles, respectively. The images showed spherical nanoparticles along with a few nanorods. The crystallite sizes of ZnO spheroidal particles were found to be ranging from 5–20 nm. ZnO nanorods were found to be ranging from 5–20 nm in width and 20–40 nm in length. Figure 6(d) showed the TEM image of the 1 at.% Pt doped ZnO nanoparticles. The ZnO particle sizes and morphology were spheroidal, hexagonal and rod-like. From the TEM studies, the average size of the hexagonal type of ZnO nanoparticles was found to be 5–20 nm. The Pt particles were not found in these Figures 6(b–f) due to the amount of doping Pt metals were small.

### 3.2. SEM Sensing Layer

**Figure 7.**
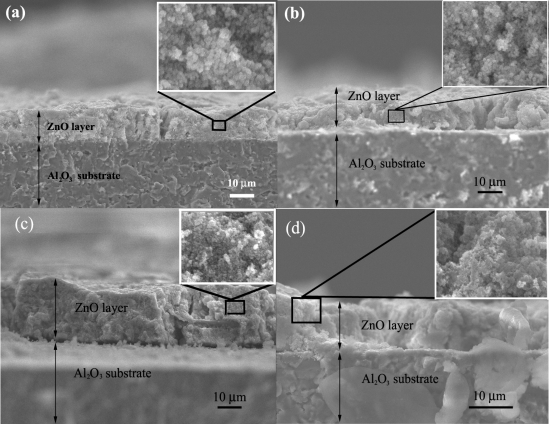
SEM micrographs of flame-made ZnO thick films as a sensor S0 (a), S1 (b), S2 (c) and S3 (d) on an Al_2_O_3_ substrate interdigitated with Au electrodes after annealing and sensing test at 350 °C in dry air. The film thickness was approximately 10 μm.

The cross-section, film thickness, and surface morphology of the sensing film layer (S0, S1, S2 and S3) after annealing and sensing test at 400 °C were observed using SEM analysis as shown in Figure 6. The film thickness of sensing film was approximately 10 μm (side view) as shown in Figure 7(a)–(d), which benefited tremendously to H_2_ gas sensing properties. Regularities in the film thickness (top view) stem from the spin coating technique. The high density Al_2_O_3_ substrate interdigitated with Au electrodes was also visible. After annealing process, a denser film layer was formed.

### 3.3. Gas Sensing Properties

**Figure 8.**
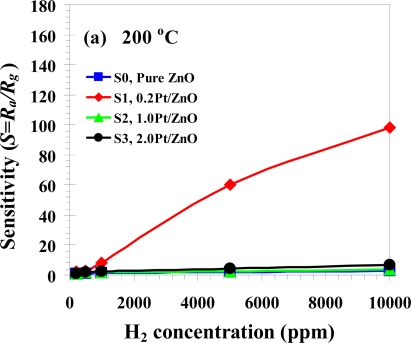

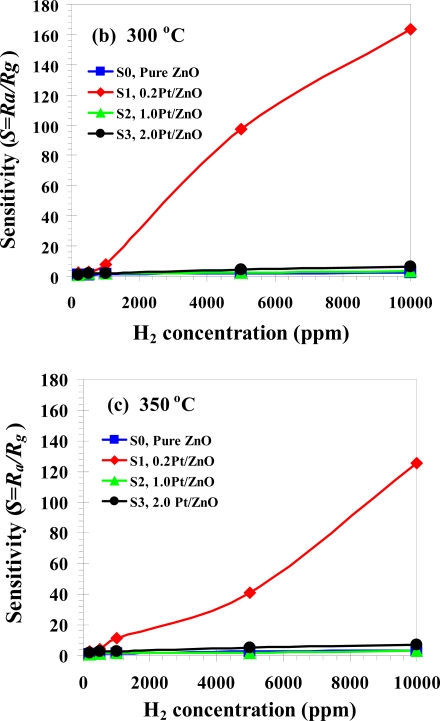
Variation operating temperature of sensitivity with concentration of H_2_ (200–1% in volume percentage of concentration) for S0, S1, S2 and S3 at 200 °C (a), 300 °C (b) and 350 °C (c).

**Figure 9.**
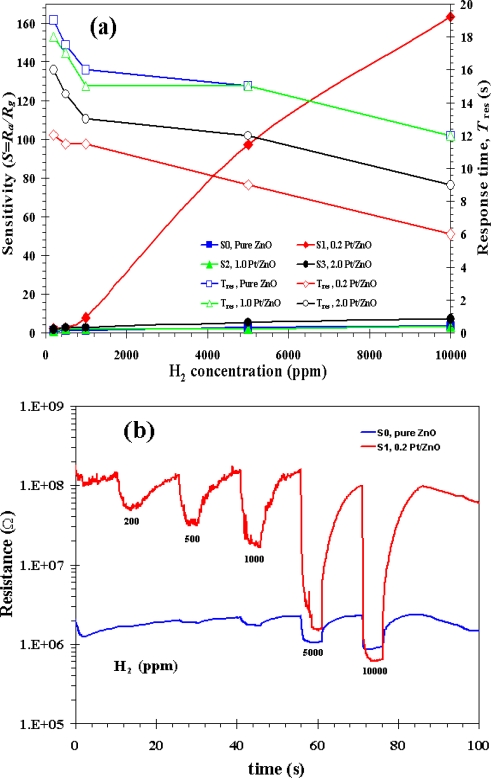
Variation of sensitivity (left) with concentration of H_2_ (0.02–1% in volume percentage of concentration) and variation of response times (right) for S0, S1, S2 and S3 (a). Change in resistance of sensor S0 and S1 (b) under exposure to reducing gas H_2_ during forward cycle.

The sensitivity and response time of the thin films of ZnO nanoparticles as a function of H_2_ concentration between 0.02 and 1% in volume percentage of concentration at 200, 300 and 350 °C are shown in Figure 8. The sensitivity increased considerably by doping the ZnO nanoparticles with 0.2 at.% Pt from 98 at 200 °C to 164 at 300 °C [show in Figures 8(a,b)]. On the other hand, the sensitivity decreased by doping the ZnO nanoparticles with 0.2 at.% Pt was 126 at 350 °C as shown in Figure 8(c). Therefore, doping the ZnO nanoparticles with 0.2 at.% Pt sensor at 300 °C had better sensitivity than 350 °C and 200 °C, respectively. In Figure 9(a) it can be seen that the sensitivity increased considerably by doping the ZnO nanoparticles with 0.2 at.% Pt. The sensitivity of 164 and response time of 6 s were obtained at 1% in volume percentage of H_2_ concentration for doping the ZnO nanoparticles with 0.2 at.% Pt. Thus, in this study 0.2 at.% Pt/ZnO showed good sensitivity for H_2_ gas as compared to the other literatures. In the present study the sensitivity, however decreased considerably by doping ZnO nanoparticles with 1.0 and 2.0 at.% Pt. It is important to note that undoped and 0.2 at.% Pt doped ZnO nanoparticles behalf as an n-type semiconductor with the resistance decreased during H_2_ gas exposure, a typical behavior of ZnO material [42]. The gas-sensing sensitivity, *S* is defined as the ratio *R_a_/R_g_*, where *R_a_* is the resistance in dry air, and *R_g_* is the resistance in test gas. The response time, *T*_res_ is defined as the time required until 90% of the response signal is reached. The recovery times, *T*_rec_ denotes the time needed until 90% of the original baseline signal is recovered.

The sensor characteristics of sensing films are affected by the film structure, morphology, and film thickness, which are determined by the film preparation procedure. In addition, the sensing temperature and film thickness are important parameters, which affect the gas sensing properties in terms of sensitivity, response, and recovery time. In gas sensors, small-sized particles decrease the response time and increase the gas sensitivity, while a low degree of agglomeration facilitates the production of thin or thick films [43], which is suitable for general gas sensing, but cannot be applied to the as-prepared high Pt% (>1%) nanoparticles. The sensitivity and response time of the thin films of ZnO nanoparticles as a function of H_2_ concentration between 0.02 and 1% in volume percentage of concentration at 200, 300 and 350 °C are shown in Figure 8. It is evident that the sensitivity of 0.2 at.% Pt doped ZnO nanoparticles increases by more than one order of magnitude compared to undoped one for all operating temperatures from 200 to 350 °C. As the operating temperature increases from 200 °C to 300 °C, the sensitivity increases from 98 to 164 [shown in Figure 8(a,b)]. However, the sensitivity decreases to 126 as operating temperatures increases further to 350 °C as shown in Figure 8(c). Therefore, 0.2 at.% Pt doped ZnO nanoparticles gas sensor had optimum sensitivity at operating temperature of 300 °C, which is lower than optimum operating temperature of undoped ZnO at around 350–400 °C. This lower optimum operating temperature is attributed to Pt catalyst’s behavior that can be best reduced hydrogen at relatively low temperature. The sensitivity of 164 and response time of 6 s were obtained at 1% in volume percentage of H_2_ concentration and 300 °C operating temperature with doping the ZnO nanoparticles with 0.2 at.% Pt. In addition, a low detection limit of 50 ppm is obtained at 300 °C operating temperature is found to be around 50 ppm. It is the concentration at which sensitivity is ∼1.1, corresponding to 10% change of resistance.

**Figure 10.**
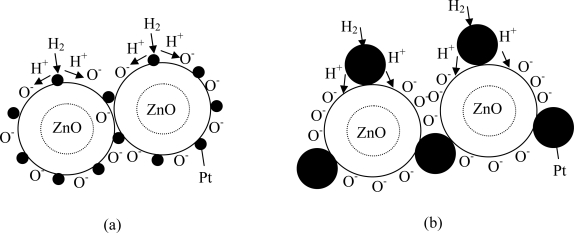
Gas sensing mechanisms based on spillover effect of Pt doped ZnO nanoparticles (a) low Pt concentration (0.2 at.%) and (b) high Pt concentration (>1 at.%).

Figure 8 show that the sensitivity was decreased considerably by doping ZnO nanoparticles with 1.0 and 2.0 at.% Pt and they were even less than undoped ZnO. The sensor behaviors versus the H_2_ concentrations ranging from 0.02 to 1% in volume percentage of concentration plot for S0, S1, S2 and S3 sensors based on an as-prepared flame-made (5/5) ZnO nanoparticles towards H_2_ gas at sensing operating temperature 300°C is shown in Figure 9(a) and changes in resistance of S0 and S1 are shown in Figure 9(b). The sensitivity as a function of the gas concentration follows the well-known power law behavior. It can be seen again that 0.2 at.% is an optimum Pt doping concentration and higher concentration significantly degrade gas sensing behavior. The semiconducting and gas-sensing behaviors are thus depending strongly on the doping level of Pt on ZnO nanoparticles. The observed gas-sensing dependence on Pt concentration may be explained as follows:

It is widely believed that Pt catalyst enhances reducing gas sensing of metal oxide via spillover mechanism [44]. This interaction is a chemical reaction by which additives assist the redox process of metal oxides. The term spillover refers to the process, illustrated in Figure 10, namely the process where the metal catalysts dissociates the molecule, then the atom can ‘spillover’ onto the surface of the semiconductor support. At appropriate temperatures, reactants are first adsorbed on to the surface of additive particles and then migrate to the oxide surface to react there with surface oxygen species, affecting the surface conductivity. For the above processes to dominate the metal oxide resistance, the spilled-over species must be able to migrate to the interparticle contact. Thus, for a catalyst to be effective there must be a good dispersion of the catalysts so that catalyst particles are available near all contacts. Only then can the catalysts affect the important interparticle contact resistance.

From the experimental results, the sensitivity of Pt/ZnO at 2at.% is lower than that at 0.2at.% but the dBET of 2 at.% Pt/ZnO nanoparticles is smaller. This can be explained from Figure 10. In Figure 10(a), it can be seen that small Pt particles for the case of low Pt concentration (0.2 at.%) can cause agglomeration so that two or more ZnO nanoparticles are connected. As a result, the low Pt concentration case will have larger average particle diameter, *d_BET_*, than high Pt concentration one [Figure 10(b)]. However, spillover effect for the case of low concentration, which has larger dBET, is considerably more effective than the other case. For low Pt concentration (0.2 at.%), Pt nanoparticles are very small compared to ZnO ones and they can be well dispersed on ZnO nanoparticles, as shown in Figure 10(a). Thus, Pt nanoparticles are very effective catalyst via spillover mechanisms. In contrast, larger Pt nanoparticles, which are formed at higher Pt concentrations, can not be well dispersed and cause possible separation among ZnO nanoparticles as shown in Figure 10(b). Therefore, catalytic action of Pt becomes considerably less effective. This is the reason why gas sensitivity significantly decreases at higher Pt concentration even though the average particle size measured by *d_BET_* is decreased at higher Pt concentration (recall that the average particle size decreases because of less agglomeration as Pt nanoparticle size increases).

**Figure 11.**
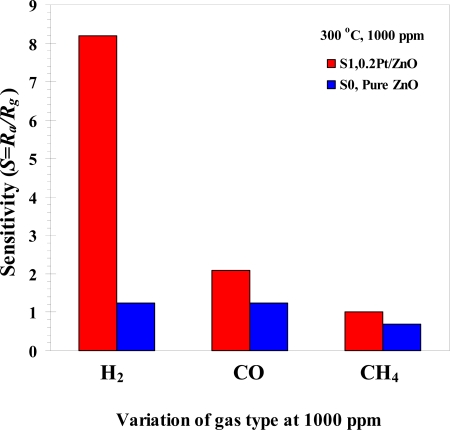
Variation of sensitivity with concentration of H_2_, CO and CH_4_ (at 300 °**C**, 0.1% in volume percentage of concentration) for sensor S0 (pure ZnO) as compared to S1 (0.2 Pt/ZnO).

The gas sensing selectivity of ZnO gas sensor has been characterized toward two other common reducing gases, carbonmonoxide (CO) and methane (CH_4_), as shown in Figure 11, which shows that 0.2 at.% Pt-doped ZnO gas sensor has a good gas selectivity for 0.1% in volume percentage of H_2_ concentration of 8.2 at 300 °C. The sensitivity of 0.2 at.% Pt-doped ZnO gas sensor of CO and CH_4_ were 2.1 and 1.0 at 0.1% in volume percentage of concentration and 300 °C operating temperature. Thus, the gas sensitivity of 0.2 at.% Pt-doped ZnO to H_2_ is higher than that to CO and CH_4_. The hydrogen selectivity of 0.2 at.% Pt-doped ZnO is also substantially higher compared than undoped ZnO gas sensor whose hydrogen sensitivity is on the same order as those of CO and CH_4_.

## 4. Conclusions

The flame-made (5/5) ZnO nanoparticles for use as H_2_ gas sensors were successfully produced by flame spray pyrolysis. The XRD patterns show that the particles correspond to hexagonal phase of ZnO and also the corresponding Al_2_O_3_, Au, and ZnO peaks were evidently seen from the flame made ZnO nanoparticles printed on Al_2_O_3_ substrate interdigitated with Au electrodes after sensing test at 300 °C as an H_2_ sensor. The *SSA*_BET_ increased and the *d*_BET_ decreased with increasing Pt concentration from 1 to 2 at.%. The morphologies of ZnO nanoparticles was observed to be mainly spheroidal particles (5–20 nm) with occasional hexagonal (5–20 nm) and rod-like particles (5–20 nm in width and 20–40 nm in length). The H_2_ sensing behaviors were found to improve with small Pt content of 0.2% but deteriorated at higher Pt concentration. The sensitivity for 1% in volume percentage of H_2_ concentration was 164 for 0.2 at.% Pt doped ZnO and at 300 °C. The response and recovery times were generally well within 6 s in the regime of high sensitivity. Thus, ZnO nanoparticles doped with 0.2 at.% Pt shows good H_2_ sensitivity at operating temperature of 300 °C. This present study showed FSP method can control the morphology, sizes, and Pt doping of nanopowders using the appropriate precursor and flame conditions. The effect of Pt loading improved H_2_ sensing behavior in terms of sensitivity, shorter response and recovery times.

## Figures and Tables

**Figure 4.**
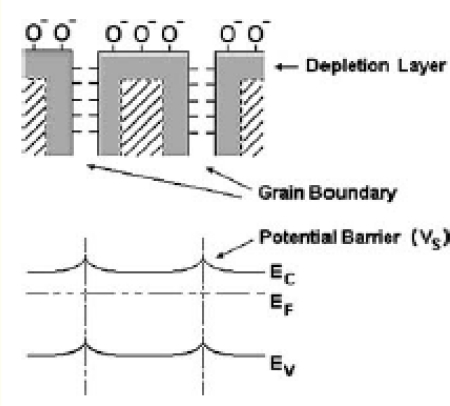
Formation of depletion layers in the surface and grain-boundary regions of ZnO due to oxygen chemisorption. The potential barriers at the grain boundaries reduce the carrier mobility.

**Table 1.** Summary on comparision of metal-doped ZnO with several synthetic methods for H_2_ sensing.

**Authors**	**Method**	**Doping level**	**Range (H_2_)**	**Sensitivity**

Xu *et al*. [6]	Chemical precipitation	0.5 wt% Pt	0.2% in air	2 at 330 °C
		0.5 wt% Ru	0.2% in air	6 at 230 °C and 2 at 400 °C
		0.5 wt% Rh	0.2% in air	115 at 300 °C
		0.5 wt% Ag	0.2% in air	9 at 400 °C

Rout *et al*. [8]	ZnO nanowires and ZnO nanotubes by electrochemical deposition	0 at.% Pt	1,000 ppm in air	43 at 150 °C
			1,000 ppm in air	
		1 at.% Pt	1,000 ppm in air	825 at 150 °C
			1,000 ppm in air	
		0 at.% Pt		18 at 150 °C
		1 at.% Pt		740 at 150 °C

Wang *et al*. [9]	ZnO nanorods deposited by Molecular Beam Epitaxy (MBE)	Pt-coated	500 ppm in N_2_	Relative response of up to 8% H_2_ at room temp.

Tien *et al*. [10]	ZnO nanorods / thin films deposited by Molecular Beam Epitaxy (MBE)	Pt-coated	500 ppm in N_2_	Response of Pt-coated nanorods was 3 times of thin films as prepared ZnO

Phanichphant *et al*. [this work]	Flame spray pyrolysis	0 at.%	1% (10,000 ppm in air)	0.2 at.%Pt showed the highest sensitivity of 164 at 350 °C
		0.2 at.%		
		1 at.%		
		2 at.%		0.2 at.%Pt showed the highest sensitivity of 8.2 at 350 °C
			0.1% (1,000 ppm in air)	
